# Supplementary material for: The Analysis of Platelet-Derived circRNA Repertoire as Potential Diagnostic Biomarker for Non-Small Cell Lung Cancer
Source: Cancers (Basel). 2021 Sep 16;13(18):4644. doi: 10.3390/cancers13184644 (PMC8468408; doi:10.3390/cancers13184644)
Supplement: Supplementary file 1 [file cancers-13-04644-s001.zip › cancers-1338892 supplementary.pdf]

cancers

MDPI

Supplementary Materials: The Analysis of Platelet-Derived circRNA Repertoire as Potential Diagnostic Biomarker for Non-Small Cell Lung Cancer

Silvia D'Ambrosi, Allerdien Visser, Mafalda Antunes-Ferreira, Ankie Poutsma, Stavros Giannoukakos, Nik Sol, Siamack Sabrkhany, Idris Bahce, Marijke J.E. Kuijpers, Mirjam G.A. Oude Egbrink, Arjan W. Griffioen, Myron G. Best, Danijela Koppers-Lalic, Cees Oudejans, and Thomas Würdinger

(a)

Sample	Type	Gender	Age	Stage	Hospital location #
Control1	control	F	67	0	1
Control2	control	M	80	0	1
Control3	control	F	63	0	1
Control4	control	F	63	0	2
Control5	control	F	63	0	2
Control6	control	M	72	0	2
NSCLC1	cancer	M	69	IV	2
NSCLC2	cancer	F	56	IV	2
NSCLC3	cancer	F	62	IV	2
NSCLC4	cancer	F	67	III	1
NSCLC5	cancer	F	62	IV	1
NSCLC6	cancer	M	80	II	1

(b)

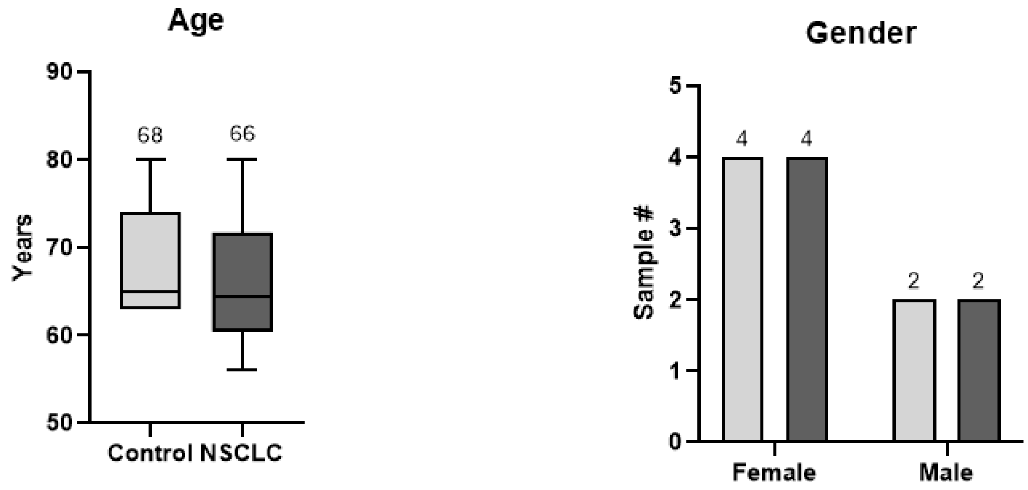

Supplementary Figure S1. Sample information of the platelet reference cohort used for circRNA analysis by RNA-sequencing. (a) Table with the general information on samples used in the study. Columns: Samples names; Type: Control refers to platelets samples derived from asymptomatic individuals and Cancer refers to samples derived from NSCLC patients; Gender: female (F) and male (M); Age: years; Stage: tumor stage at the time of the blood withdrawal; and Hospital location #: blood samples were windrowed in two different hospitals: Maastricht University Medical Center (1) and VU University Medical Center, Amsterdam (2). (**b**) Control and NSCLC group were balanced per age and gender. Average age per group and the number of samples per gender are indicate above graphic bars.

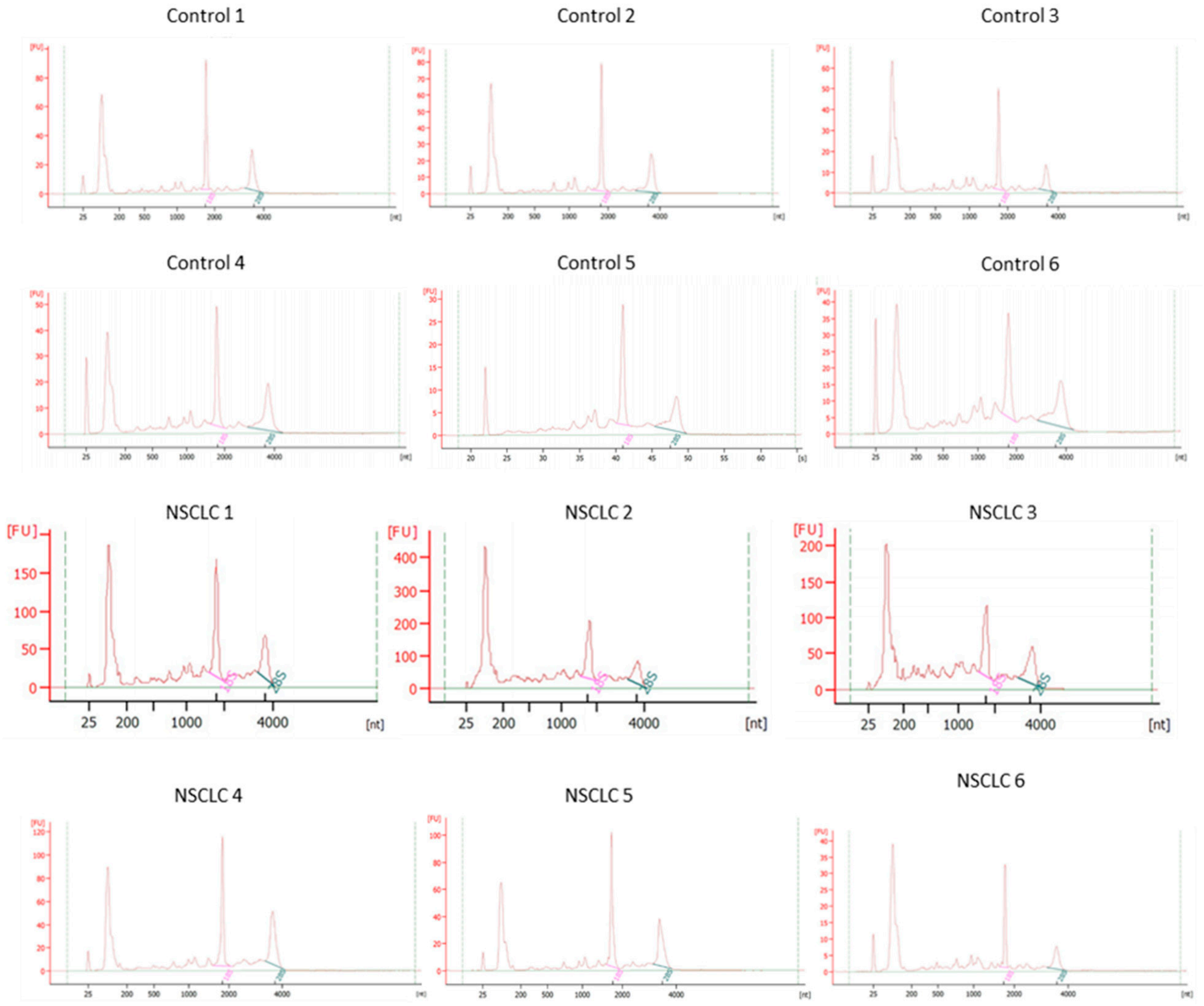

Supplementary Figure S2. Overview of Bioanalyzer profiles of RNA extracted from platelets. Quality control and quantification of the total RNA derived from platelets by Agilent Bioanalyzer Picochip analysis. Images are representative for other Bioanalyzer results.

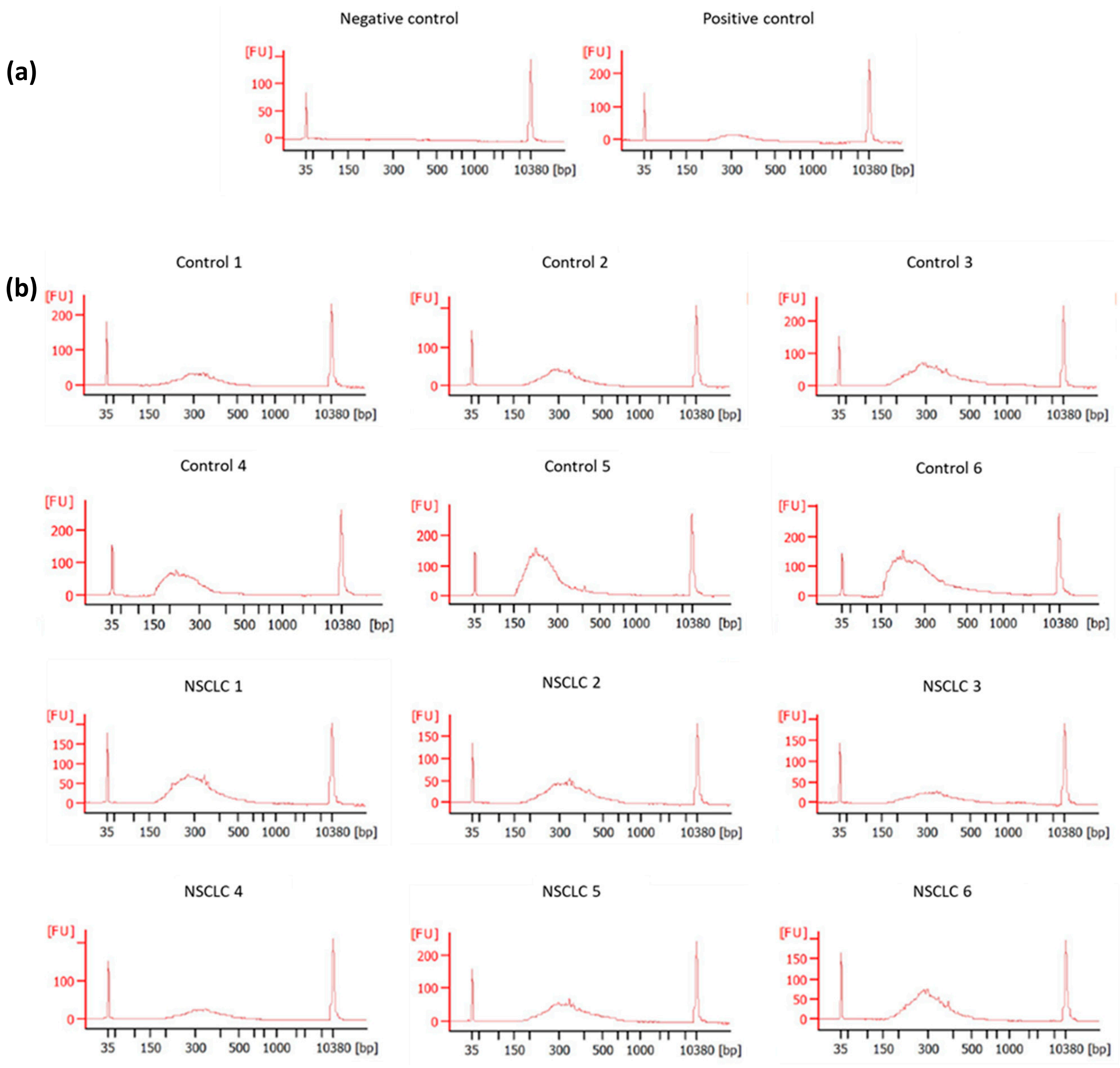

Supplementary Figure S3. Bioanalyzer profiles of the cDNA libraries. Platelets RNA-sequencing libraries analysis by Agilent Bioanalyzer DNA 7500. (**a**) Negative and positive controls subjected to SMARTer cDNA amplification and library preparation. The negative control should show no signal. Positive control should show a signal in the region between 150 bp and 750 bp. (**b**) Bioanalyzer profiles of the 12 platelets samples used for the RNA-sequencing experiment.

(a)

Sample	Type	Gender	Age	Stage	Hospital location #	Histological type	Smoking
CNTR1	control	M	58	0	1	N/A	0
CNTR2	control	F	64	0	1	N/A	0
CNTR3	control	M	56	0	1	N/A	1
CNTR4	control	F	52	0	1	N/A	2
CNTR5	control	M	60	0	1	N/A	1
CNTR6	control	F	52	0	1	N/A	0
CNTR7	control	F	65	0	1	N/A	0
CNTR8	control	F	59	0	1	N/A	1

CNTR9	control	M	52	0	1	N/A	1
CNTR10	control	M	64	0	1	N/A	0
CNTR11	control	F	61	0	1	N/A	0
CNTR12	control	F	56	0	1	N/A	0
CNTR13	control	F	66	0	1	N/A	0
CNTR14	control	M	53	0	1	N/A	0
CNTR15	control	M	61	0	1	N/A	N.A.
CNTR16	control	F	64	0	1	N/A	0
CNTR17	control	F	68	0	1	N/A	2
CNTR18	control	M	65	0	1	N/A	1
CNTR19	control	M	60	0	1	N/A	2
CNTR20	control	M	73	0	1	N/A	2
CNTR21	control	M	79	0	1	N/A	2
CNTR22	control	M	68	0	1	N/A	2
CNTR23	control	M	79	0	1	N/A	0
CNTR24	control	M	75	0	1	N/A	2
NSCLC_P1	cancer	F	72	IV	2	A	1
NSCLC_P2	cancer	M	46	IV	2	A	1
NSCLC_P3	cancer	M	57	IV	2	A	1
NSCLC_P4	cancer	M	54	IIa	1	S	1
NSCLC_P5	cancer	M	68	Ib	1	A	1
NSCLC_P6	cancer	F	53	Ia	1	A	2
NSCLC_P7	cancer	F	65	Ia	1	A	2
NSCLC_P8	cancer	F	59	Ia	1	O	2
NSCLC_P9	cancer	F	70	Ia	1	O	1
NSCLC_P10	cancer	M	60	IIIa	1	S	2
NSCLC_P11	cancer	M	67	IIIa	1	O	1
NSCLC_P12	cancer	F	52	Ib	1	A	0
NSCLC_P13	cancer	M	60	Ib	1	S	2
NSCLC_P14	cancer	M	78	IIIa	1	S	0
NSCLC_P15	cancer	M	71	IV	2	O	1
NSCLC_P16	cancer	F	41	IV	2	A	0
NSCLC_P17	cancer	F	44	IV	2	O	2
NSCLC_P18	cancer	M	63	IV	2	A	0
NSCLC_P19	cancer	M	67	IV	2	S	0
NSCLC_P20	cancer	M	55	IV	2	A	1
NSCLC_P21	cancer	F	77	IV	2	O	N.A.
NSCLC_P22	cancer	M	45	IV	2	O	1
NSCLC_P23	cancer	M	61	IV	2	S	2

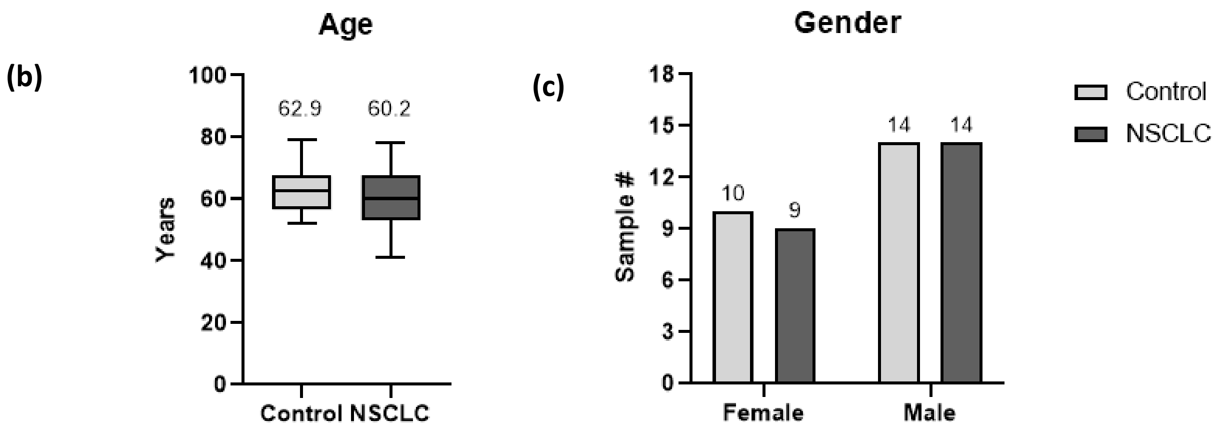

Supplementary Figure S4. Validation sample information of the platelet reference cohort used for circRNA analysis by RT-qPCR. (**a**) Table with the general information on the samples from the validation cohort. Columns: Samples ID; Samples names; Type: Control refers to platelets samples derived from asymptomatic individuals and Cancer refers to samples derived from NSCLC patients; Gender: female (F) and male (M); Age: years; Stage: tumor stage at the time of the blood withdrawal; Hospital location #: blood samples were collected in two different hospitals: Maastricht University Medical Center (1) and VU University Medical Center, Amsterdam; Histological type are divided in: (A) adenocarcinoma, (S) squamous cell carcinoma, (O) other histological types or histological data not available, and (N/A) not applicable; Smoking: (0) no smoker, (1) smoker, (2) formal smoker and (N.A.) data not available. Control and NSCLC group were balanced per age (**b**) and gender (**c**).

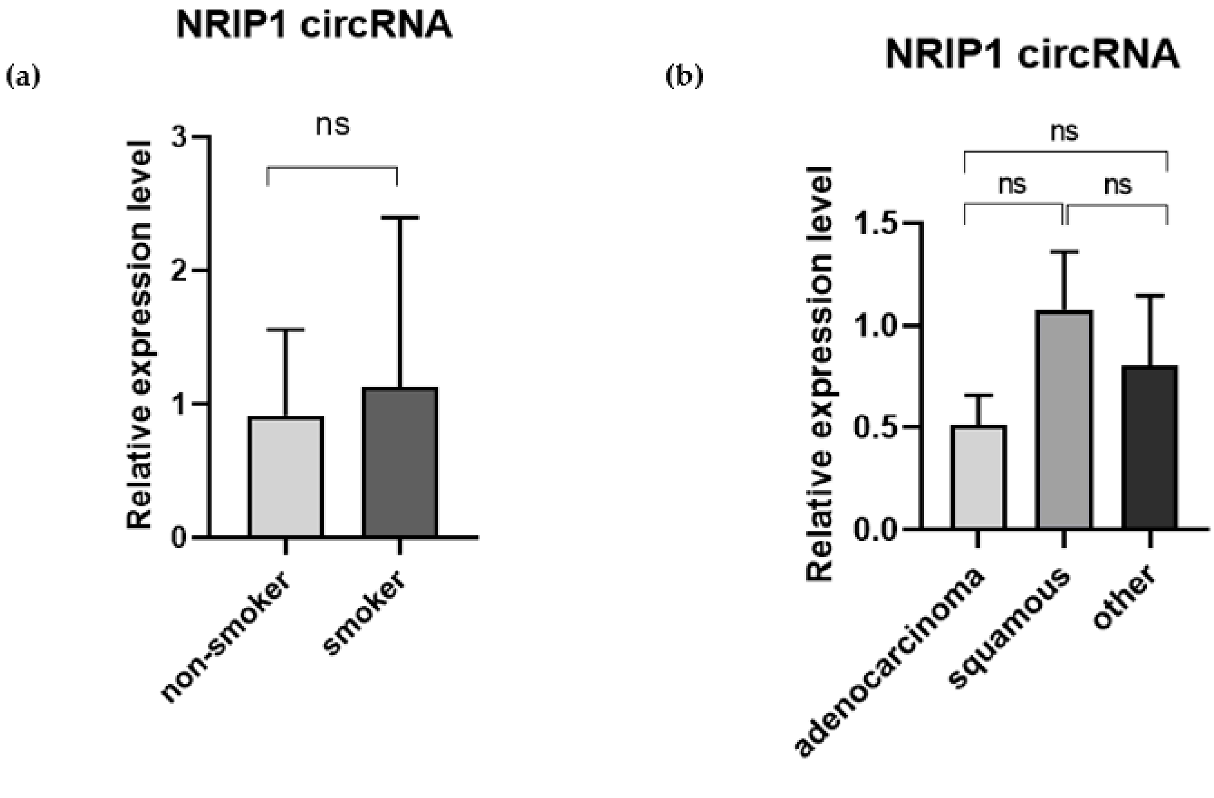

Supplementary Figure S5. circNRIP1 expression in platelet-derived RNA by RT-qPCR evaluated by histological type and smoking habits. The expression of circRNA NRIP1 and MAN1A2 were analyzed by RT-qPCR. The 2^-ΔΔCt method was used to calculate the relative expression of circNRIP1 between the different groups. For the statistical analysis we used the unpaired nonparametric t-test with Welch correction with 2-tailed P-value. (**a**) Samples are divided in (formal) smokers (n=28) and non-smokers (n=17). No significant difference (indicated as ns) was observed on the expression of circNRIP1

(p-value=0.4674). (**b**) Platelet samples derived from NSCLC patients were divided in three histological subtypes: patients diagnosed with adenocarcinoma (adenocarcinoma, *n*=10); patients diagnosed with squamous cell carcinoma (squamous, *n*=6); patients with other histological variants or no histological data available (other, *n*=7). The expression level of circN-RIP1 shows no significant differences (indicate as ns) between adenocarcinoma and squamous group (*p*-value=0.1164), between adenocarcinoma and others subtypes (*p*-value=0.4497) and between squamous cell carcinoma and other subtypes (*p*-value=0.5535).

Supplementary Table S1. Summary table of the primer design. The chromosomal region corresponds to the back splicing junctions of circRNA NRIP1 (target of interest) and circRNA MAN1A2 (control "housekeeping" circRNA). Primers were designed to amplify the back splicing region of the two circRNAs. Probes were labeled with 5′-6FAM and 3′-DQ.

	**circNRIP1**	**circMAN1A2**
**Back-splicing junction (hg38)**	chr21:15014344-15043574	chr1:117402186-117420649
**Forward primer (5′-3′)**	TGAAGGAG-GACAGGGAATCTGAA	CGATTTATTGGAGGCCTACTTGC
**Reverse primer (5′-3′)**	GGCTGTGTTTCTCCCAAATGTT	TCTGGGTCTCCACCACGTAT
**Probe (6-FAM + DQ)**	AGAGGCTCAGAGCTTGGAGA-CAGAC	GCCACCAGTCCCTATTCCCAAC-CTTGT
**Concentrations**	600/300/250 nM	300/600/250 nM

Supplementary Table S2. ANOVA output of the top 30 circRNAs (out of 411) differentially expressed in platelets of NSCLC patients (*n*=6) versus platelets of asymptomatic individuals (*n*=6), ordered by increasing p-value. The origin of the circRNAs is provided by chromosome number followed by position of back-splice junctions (GRCh38/hg38), positive (+) or negative (−) strand and size of genomic region. LogFC: logarithm of fold change, logCPM: logarithm of counts per million, LR: likelihood ratio, *p*-value: probability value, FDR: *p*-value corrected for false-detection rate.

**circRNA**	**Chr_position**	**logFC**	**logCPM**	**LR**	**P-value**	**FDR**
NRIP1	chr21:15043574-15014344	-0.29592	16.15014	38.16888	6.49E-10	3.07×10^{6}
RNF19B	chr1:32949774-32948222	-5.00585	6.827754	12.68557	0.000368	0.408548
AMD1	chr6:110887505-110888983	-5.26108	6.960953	12.52172	0.000402	0.408548
SH3BGRL	chrX:81276984-81298357	-4.59651	7.231869	12.43663	0.000421	0.408548
ST7L	chr1:112616895-112597971	-4.63557	7.255242	12.26218	0.000462	0.408548
SUV39H2	chr10:14896846-14897517	-4.09755	7.631399	11.59124	0.000663	0.408548
RBM33	chr7:155706860-155718443	-4.1555	7.411723	10.87308	0.000976	0.408548
NO-NAME	chr2:190568073-190573419	-5.45329	7.070343	10.8723	0.000976	0.408548
KRIT1	chr7:92234923-92225720	-4.70355	6.682802	10.74499	0.001046	0.408548
PECAM1	chr17:64352463-64348260	-4.53681	6.602994	10.67477	0.001086	0.408548
AFF1	chr4:87084120-87091829	-4.61285	7.239943	10.59121	0.001136	0.408548
KIF2A	chr5:62347130-62355254	-2.86466	8.606843	10.35752	0.001289	0.408548
LINS1	chr15:100565265-100564692	-2.2521	10.04339	10.33537	0.001305	0.408548
PPA2	chr4:105424322-105396249	-3.87751	7.618442	10.28956	0.001338	0.408548
DENND4A	chr15:65706224-65702305	-4.15537	6.457191	10.08241	0.001497	0.408548
DLG1	chr3:197119530-197104903	4.554137	7.56337	9.842434	0.001705	0.408548
MYCBP2	chr13:77233263-77224451	-5.2288	6.944568	9.837442	0.00171	0.408548
RHOF	chr12:121781192-121780872	-1.68676	9.327772	9.774111	0.00177	0.408548
HTT	chr4:3086939-3107423	-5.1264	6.8903	9.564129	0.001984	0.408548
STXBP3	chr1:108752257-108760085	-5.0624	6.856096	9.42589	0.002139	0.408548
SUZ12	chr17:31988320-31988497	-3.52316	7.955087	9.353843	0.002225	0.408548
UBAP2	chr9:33973237-33971651	-4.1397	7.408808	8.983093	0.002725	0.408548
NCOR1	chr17:16158873-16149451	-4.80201	6.730626	8.874144	0.002892	0.408548

BRWD1	chr21:39274472-39269899	-3.19201	8.11097	8.806127	0.003002	0.408548
XRN2	chr20:21339044-21344208	-4.056	6.926366	8.801219	0.00301	0.408548
DCAF12	chr9:34098517-34096716	-3.76483	7.504243	8.705537	0.003172	0.408548
USP32	chr17:60294801-60265412	-4.32634	7.070119	8.66162	0.00325	0.408548
FANCL	chr2:58232112-58198579	-4.63002	6.650712	8.584887	0.00339	0.408548
PRR5L	chr11:36393846-36403378	2.698679	8.433516	8.562247	0.003432	0.408548
VAC14	chr16:70763025-70762540	-4.1447	6.97075	8.553315	0.003449	0.408548
